# Design and validation of A 30 ghz 8 × 8 slot antenna array with ridge waveguide pins/holes layers

**DOI:** 10.1038/s41598-025-91583-y

**Published:** 2025-03-11

**Authors:** Hasan Raza, Slawomir Koziel, Stanislaw Szczepanski

**Affiliations:** 1 Department of Engineering, Reykjavik University, 102 Reykjavík, Reykjavik, Iceland; 2 Faculty of Electronics, Telecommunications and Informatics, Gdansk University of Technology, Gdansk, 80-233 Poland

**Keywords:** Ridge waveguide, Hollow waveguide, Slot antennas, Electromagnetics, Propagation, Electrical and electronic engineering, Electronic and spintronic devices

## Abstract

This paper presents an 8 × 8-element slot antenna array optimized for 30 GHz band applications, achieving high gain, wide impedance bandwidth, and high efficiency. The array employs a pin/hole-based design, which enables a compact structure and reduces fabrication complexity and cost, as it eliminates the need for electrical contact between its three primary layers: the metal radiating slot plate, a sub-array cavity layer, and a ridge waveguide feed network layer. The corporate feed network is realized through an array of pins and guiding ridges integrated into a metal plate, effectively distributing power to the radiating elements. A double transition from ridge waveguide to rectangular waveguide, leading to a 2.92 mm coaxial connector, ensures efficient feeding. Each component, including the radiating elements, cavity layer, power dividers, and transitions, is designed and optimized to maintain a low reflection coefficient (|*S*_11_| < -10 dB) across the 25–35 GHz frequency range. The 8 × 8 array is fabricated using standard milling techniques. The measured impedance matching bandwidth of approximately 33% is obtained, covering the entire 25-to-35 GHz range. The array consistently demonstrates a gain of over 23 dBi validating its performance for high-frequency applications.

## Introduction

The growing demand for higher data throughput in modern communication systems has driven the satellite communications industry toward higher frequency bands, including the Ka-band with the development of high throughput satellites. The Ka-band offers several advantages, including greater spectral availability and the implementation of spot-beam coverage, enabling efficient frequency reuse^1^. These attributes make the Ka-band particularly attractive for emerging applications, such as satellite communication systems.

In response to these demands, there has been significant research dedicated to developing compact, lightweight, and cost-effective antennas suitable for Ka-band applications^2^. Despite this progress, achieving antenna designs that combine high efficiency, dual-band capability, low profile, and affordability remains a challenge. Planar array antennas offer several advantages compared to reflector and lens antennas in terms of their compact size, ease of assembly, and enhanced aperture efficiency^3^. Various efforts have been dedicated to the development of high-gain array antennas with improved efficiency. The Magneto-Electric (ME) dipole array antenna represents an advanced design that integrates electric and magnetic dipoles to deliver enhanced performance in terms of broad bandwidth, stable radiation patterns, and high polarization purity^4^. Despite these advantages, the ME dipole array has limitations that must be carefully evaluated for specific applications. Among these, the requirement for a complex feeding network to achieve uniform excitation and phase coherence across array elements poses a significant design and implementation challenge^5^. Waveguide slot antenna arrays stand out as prime contenders for high-gain wideband planar antenna applications across various fields. These antennas exhibit minimal susceptibility to both dielectric and radiation losses, making them well-suited for scenarios demanding high gain and efficiency^6^. Nevertheless, achieving wideband functionality with waveguide slot arrays necessitates complicated corporate feed networks, which can lead to complexities and bulkiness^7^. Moreover, when operating at higher frequencies, these feed networks demand careful design, high-precision, and costly manufacturing processes. Ensuring effective electrical connections between various metal layers within the structure proves particularly challenging. In pursuit of this goal, techniques such as microstrip configurations^8,10^and substrate integrated waveguides (SIW)^11,13^have been explored. Nevertheless, these methods suffer from significant losses, particularly limiting their viability in applications involving high frequencies, such as the Ka-band and beyond. SIWs offer a compact structure and cost-effective manufacturing, enabling complete circuit integration into a single plate^14,15^. However, guiding waves within a dielectric slab is associated with increased losses, especially at higher frequencies. On the contrary, hollow waveguides exhibit superior performance in terms of efficiency and high power handling^16^. Unfortunately, establishing proper electrical connectivity among various metal components is a costly and complicated endeavor, thereby restricting the practical implementation of waveguide-based high-gain slot arrays, especially at higher frequencies. In the effort to achieve robust electrical connections, the strategy of diffusion bonding thin copper plates has been adopted in studies, leading to the development of corporate-fed hollow waveguide array antennas.

Another technique, a gap waveguide, guides waves in air, reducing losses compared to dielectric-based SIW^17,19^. Gap waveguides eliminate the need for tight metallic contacts between the top and bottom plates, such as in hollow waveguide, facilitating the manufacturing process. They also prove beneficial in packaging by minimizing unwanted leakage, as well as reduce cavity resonances. The most common type of gap waveguide is the pin-type electromagnetic bandgap (EBG) structure, which effectively suppresses wave propagation in undesired areas. Yet, this technology exhibits its own challenges, requiring the manufacturing of thin, long metallic pins. This raises both design costs and manufacturing complexity^20^. Efforts have been made to address these challenges, including the proposal of half-height pins to simplify pin surface fabrication^21,22^. Despite these advancements, a precision of the manufacturing process remains critical. Another cost-effective method to manufacture complex waveguide structures at high frequencies are the glide symmetric holey EBG structures^23,24^. This technique only involves holes, which makes the manufacturing process much easier as compared to manufacturing of pins. However, the periodicity of the holey EBG unit cell is about 2.5 times larger than that of the conventional pins at the same frequency, making the circuit bulkier.

A practical approach to minimizing RF leakage in millimeter-wave applications is the use of a metallic multi-layer wall with pin/hole structures^25^, which serves as an effective shielding mechanism for adjacent transmission lines on the principles of split-block configurations^26^. This method employs a series of stacked thin metal plates, creating a compact, air-filled waveguide structure. Unlike gap waveguide or holey electromagnetic bandgap (EBG) solutions, which require significant spacing between pins or holes to maintain effective isolation, this design achieves over 30 dB isolation using a single, continuous line of pins or holes embedded directly into the waveguide sidewalls. This arrangement enhances the compactness of the waveguide assembly and significantly reduces fabrication complexity by eliminating the need for strict alignment or electrical contact between the layers. Recent advancements demonstrate that such multi-layer pin/hole structures are feasible for high-frequency waveguide applications, offering a space-efficient alternative to traditional gap waveguides and glide-symmetric holey EBG structures with comparable shielding performance. Experimental validations have further confirmed the effectiveness of this configuration^25^ in maintaining high isolation and efficient performance in complex waveguide arrays, thus paving the way for cost-effective, scalable designs for next-generation millimeter-wave systems.

In this paper, we propose a simple 8 × 8 metallic slot array using multi-layer pin / hole wall as a shielding mechanism to reduce RF leakage along the line, suitable for millimeter-wave applications. The paper is organized as follows: The detailed development of a wideband 2 × 2 planar slot array operating in the Ka band is presented in Section II. Each 2 × 2 slot array is fed by a ridge waveguide underneath. The antenna design is optimized for a consistent broadside beam configuration across a 30-percent relative bandwidth, utilizing an air-filled cavity as its foundation. The cavity is subsequently excited by a T-shaped ridge located beneath it. Section III of the paper encompasses the design processes for a wideband power divider, a 90^o^ bend and the final component of the antenna array, a double transition from the ridge waveguide to a rectangular waveguide and to a 2.92 mm coaxial connector. Section IV revolves around the design of the complete 8 × 8-element array, while Section V presents and analyses the measurement results. Finally, Section VI provides a summary and conclusions.

In the context of a broad-spectrum antenna, the reflective properties (|S_11_|) of each constituent element within the antenna play a pivotal role in averting interference maxima across the complete (|S_11_|) spectrum over the entire frequency range of interest. The measured outcomes show an impressive 33% impedance matching bandwidth (|S_11_| < − 10 dB) across the 25-to-35 GHz frequency range. Moreover, the measured gain surpasses 23 dBi throughout this band.

Several original components and technical contributions of this work should be emphasized. The major innovation of the proposed structure lies in the introduction of a pin-hole structure along the waveguide. This approach effectively mitigates leakage through the joints and layers of the multi-layered waveguide structure. Additionally, it enables the design of circuits without the need for periodic structures, thereby reducing the overall area and volume required in the design. While this approach bears some resemblance to that of a substrate integrated waveguide (SIW), it fundamentally differs in that wave propagation occurs in air rather than through a dielectric medium. As a result, it effectively eliminates dielectric losses.

**Table 1:** Important design parameters of 2 × 2 element array.

Parameters	Value [mm]	Parameters	Value [mm]
*S*_*w*_	2.97	*C*_*h*_	2.4
*S*_*l*_	5.9		
*S*_*x*_	2.02	*C*_*l*_	6.23
*S*_*y*_	6.2	*C*_*w*_	3.1
*Slot Thickness*	0.5	*r*_*l*_	5.75
*Cw*_*x*_	0.97	*r*_*w*_	1.82
*Cw*_*y*_	2.82	*r*_*s*_	2.38

## Design of 2 × 2 element subarray

**Fig. 1:**
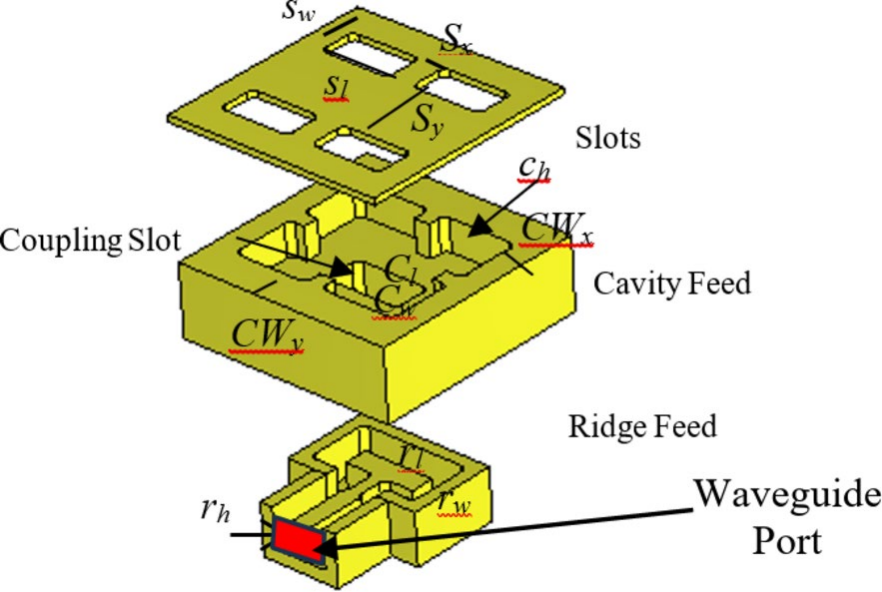
Exploded view of the 2 × 2 element sub-array.

**Fig. 2:**
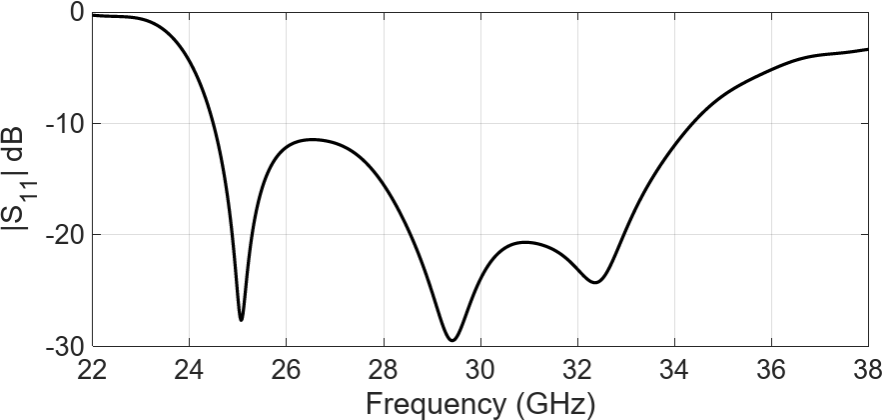
Simulated reflection coefficient of the 2 × 2 element subarray.

**Fig. 3:**
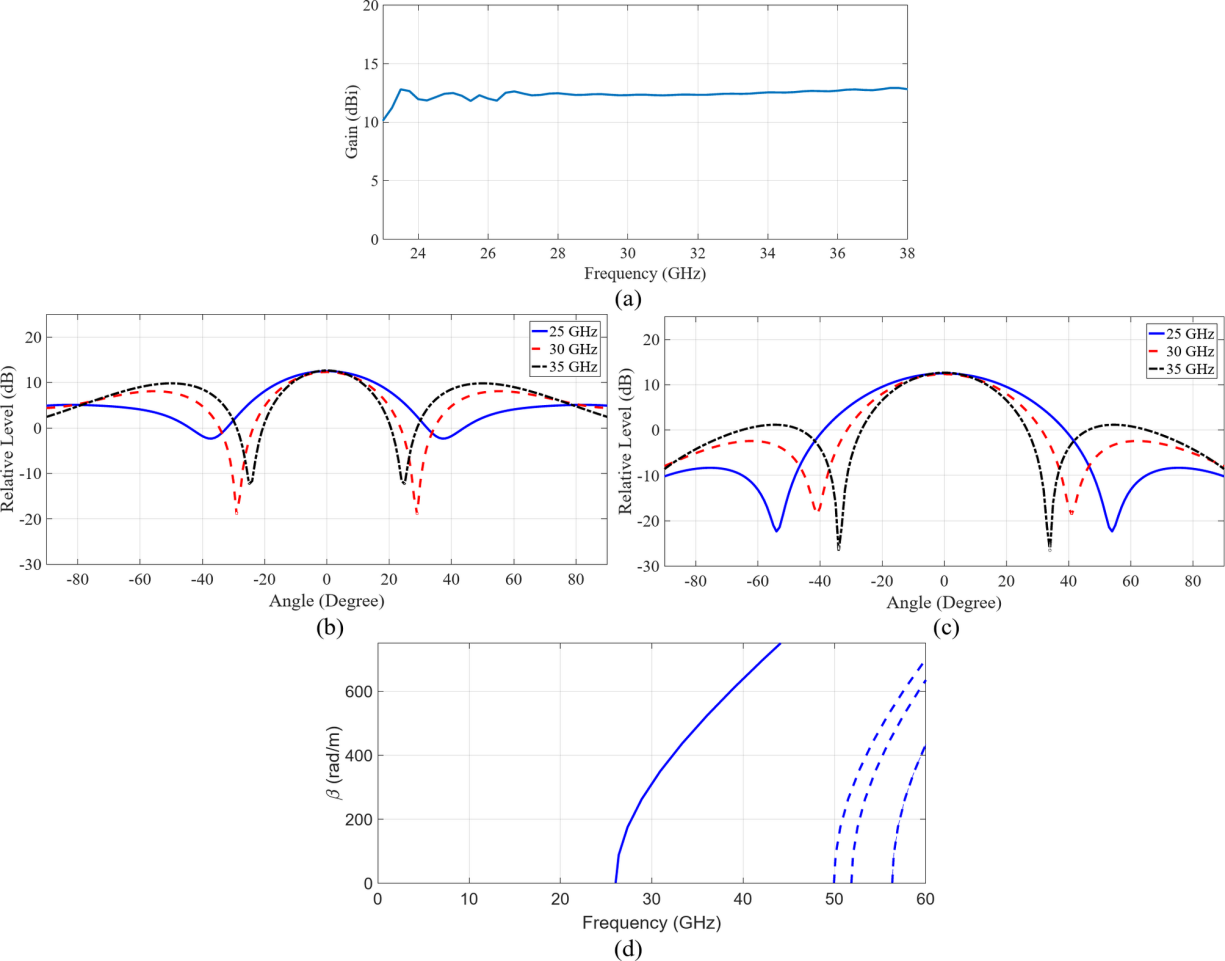
Simulated gain and radiation pattern of 2 × 2 element subarray (**a**) Gain, (**b**) E-Plane pattern, (**c**) H-Plane pattern and (**d**) modes analysis of the slot.

A configuration of the 2 × 2-element sub-array, comprising three sub-sections has been shown in Fig. 1. The bottom section consists of the feeding component, which will be further extended into a corporate feeding network comprising power dividers and bends. The middle section is the coupling slot and the cavity feed, whereas the upper section hosts the radiating slots. The feeding component is based on the ridge waveguide. Within the subsequent layer, a custom-designed coupling slot in the metal plane stimulates the cavity. The radiating part involves four slots arranged in both directions as a 2 × 2 formation on a metal plane. A precisely-designed cavity ensures equal excitation, in terms of the amplitude and phase, across these four slots, resulting in a broadside beam. Table 1outlines the important design parameters for the 2 × 2-element sub-array. Full-wave simulations of the designed sub-array were conducted utilizing CST Microwave Studio^27^. The simulated reflection coefficient, presented in Fig. 2, indicates an approximate 33% bandwidth for the reflection coefficient (|S11| < − 10 dB) within the frequency band from 25 GHz to 35 GHz. The simulated gain, approximately 12 dBi across the entire frequency range, is presented in Fig. 3(a). Additionally, the simulated radiation patterns in the E-plane and H-plane at three distinct frequencies are illustrated in Fig. 3(b) and Fig. 3(c), respectively. Moreover, the operational modes across the entire bandwidth for the given slot width and height have been analyzed, with the results summarized in Fig. 3(d). Higher-order modes begin to appear beyond 50 GHz.

## Power divider, 90-degree bend and transition

**Fig. 4:**
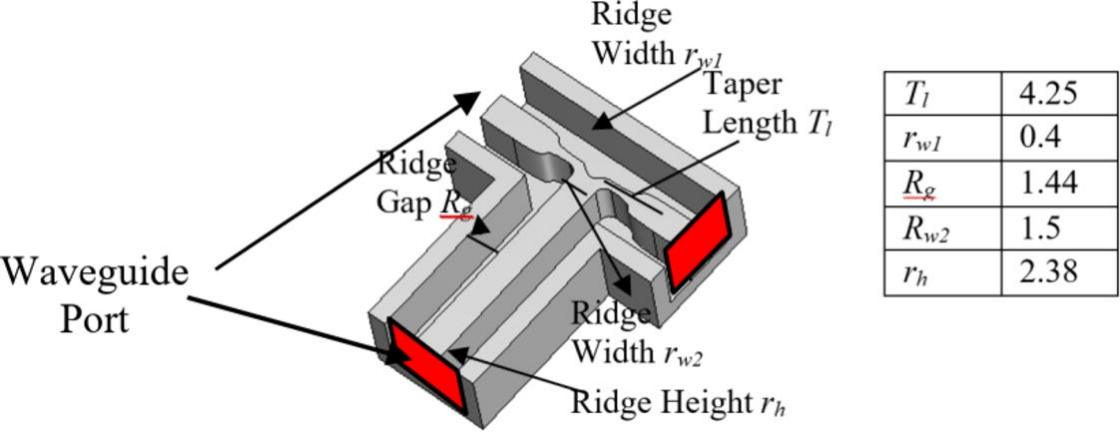
Power splitter and its parameter values in [mm].

**Fig. 5:**
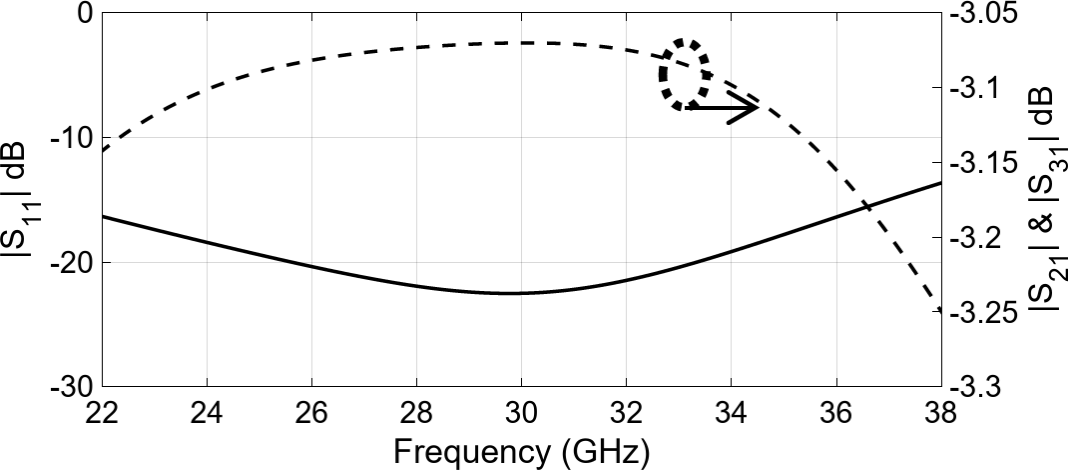
Simulated reflection and transmission coefficient of power splitter shown in Fig. 4.

**Fig. 6:**
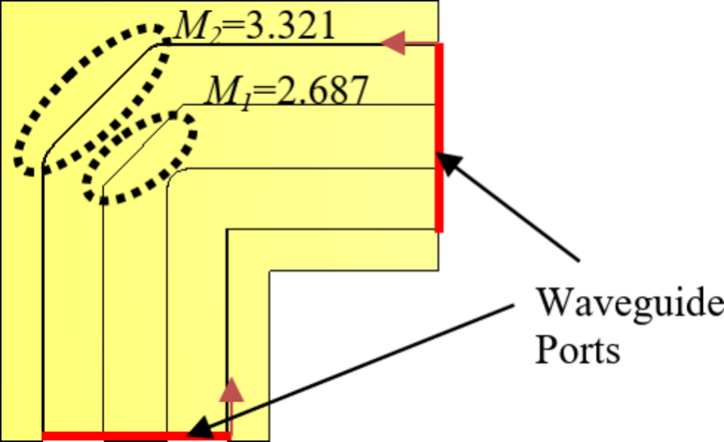
90-degree bend with its important parameters in [mm].

**Fig. 7:**
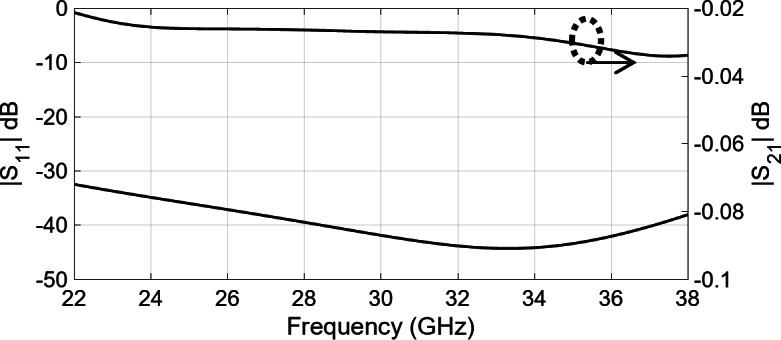
Simulated reflection and transmission coefficient of 90^o^ bend shown in Fig. 6.

To expand the intended sub-array into a larger structure, a corporate feed network is essential, incorporating power dividers and 90-degree bends. Figure 4 shows the arrangement of a two-way ridge waveguide power divider. The air gap between the top metal plate and the ridge is 0.62 mm. Notably, the output ports exhibit consistent amplitude and phase owing to the inherent symmetry. The simulated reflection coefficient, shown in Fig. 5, demonstrates excellent reflection response and a well-balanced transmission coefficient. Consequently, using the ridge waveguide technology, this power divider serves as a robust foundation for expanding into a larger corporate feeding network. Performance of the corporate feed network also depends on a 90-degree bend, as shown in Fig. 6. Through careful calibration of the mitered bend values, we have managed to attain |S_11_| of approximately − 40dB, as depicted in Fig. 7.

**Fig. 8:**
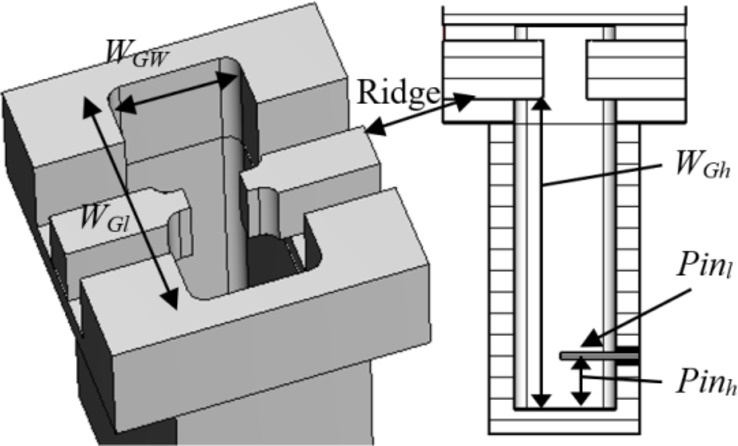
Geometry of the rectangular-waveguide-to-ridge-waveguide transition and the coaxial connector.

**Fig. 9:**
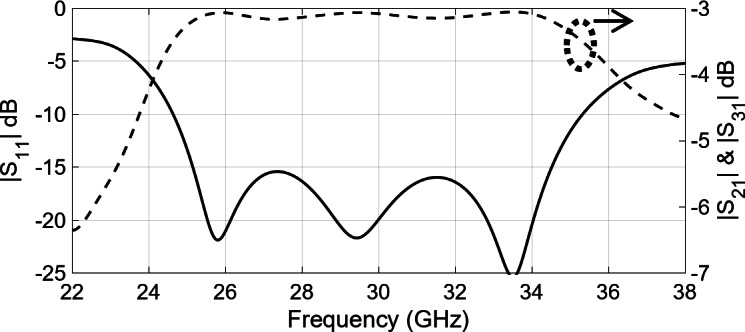
Simulated reflection and transmission coefficient of rectangular-waveguide-to-ridge-waveguide transition and the coaxial connector transition shown in Fig. 8.

Figure 8 illustrates the configuration of a straightforward E-probe rectangular-waveguide-to-ridge-waveguide transition, implemented to align the ridge waveguide line with the rectangular waveguide. Additionally, for streamlined compatibility between the antenna inputs and the measurement tools, a 2.92 mm coaxial-connector-to-rectangular-waveguide transition has been included as well. With the fine tuning of the variables shown in Fig. 8, the simulated S-parameters shown in Fig. 9 reveal the reflection level below − 15 dB across the 25–35 GHz frequency band. Furthermore, utilizing aluminum as the material, the simulated insertion loss remains under 0.06 dB within the same frequency range.

## Design of complete 8 × 8 element array

**Fig. 10:**
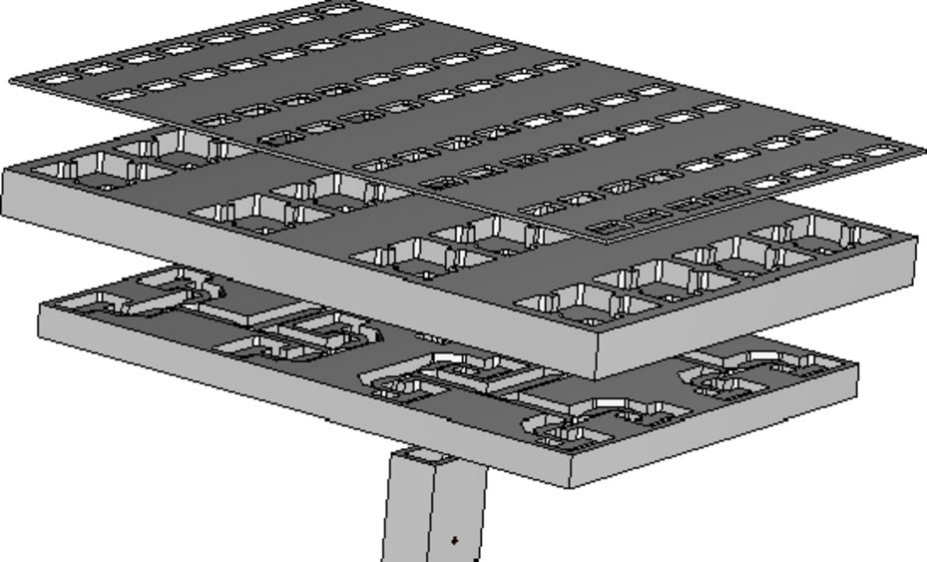
Proposed corporate-fed 8 × 8 slot array antenna.

The diagram in Fig. 10 illustrates the layout of an 8 × 8 slot array antenna. Each cavity within this array stimulates four radiating slots. To effectively feed the 16 cavities, a ridge waveguide feeding network has been engineered beneath the structure. This network ensures that each cavity receives equal amplitude and phase. The ridge waveguide network is designed to receive vertical feeding from a rectangular waveguide including a coaxial connector positioned at the center. This setup creates symmetry within the ridge waveguide network, leading to half of the slots being fed with a 180° phase offset. Symmetric feeding enables simulations on just half of the array, drastically reducing the computational time and memory usage in full-wave simulations using CST Microwave Studio.

**Fig. 11:**
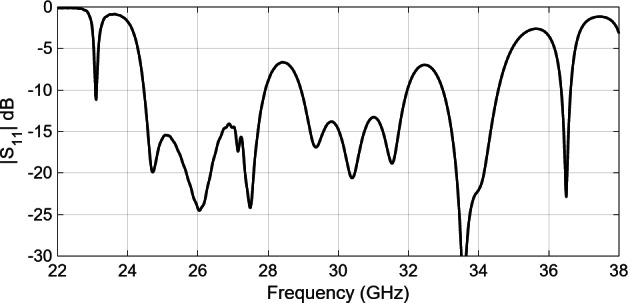
Simulated reflection coefficient of whole 8 × 8 slot array antenna.

Figure 11 shows the simulated reflection coefficient of the complete 8 × 8 slot array antenna. The results reveal that |S_11_| remains below − 10 dB across most frequencies, with exceptions around 29 and 33 GHz. This deviation could be attributed to the independent design of components outlined in Sections II and III. To achieve constructive combination and alignment of these independently designed elements within the array, the entire structure has been rigorously optimized. Here, a trust-region (TR) gradient-based algorithm^28^implemented in Matlab has been utilized with antenna response Jacobian matrix estimated using finite differentiation (FD)^29^.

The TR sub-problem has been solved using sequential quadratic programming (SQP) algorithm implemented in fmincon routine of Matlab’s Optimization Toolbox. In order to accelerate the process, a rank-one Broyden formula^30^ has been utilized to update the sensitivity matrix instead of FD when close to convergence.

**Fig. 12:**
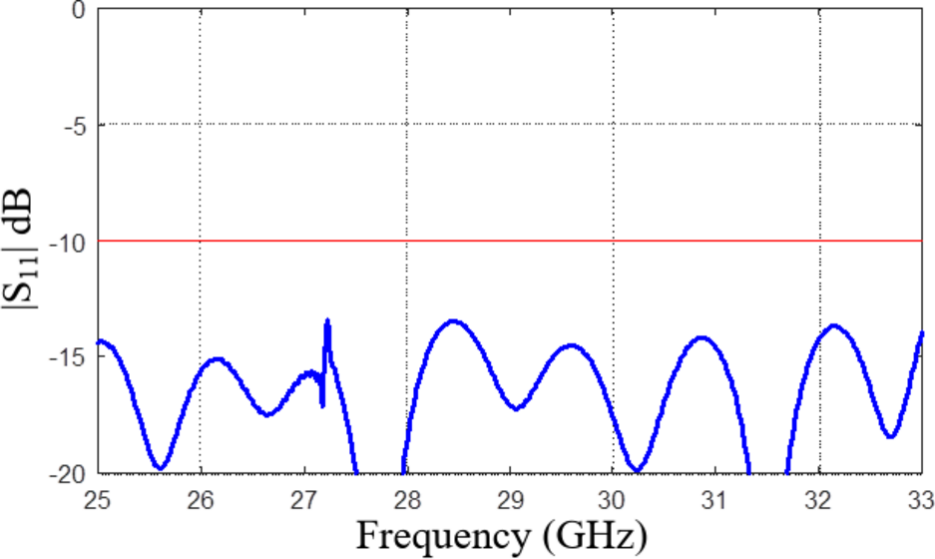
Simulated reflection coefficient of optimized 8 × 8 slot array antenna.

**Fig. 13:**
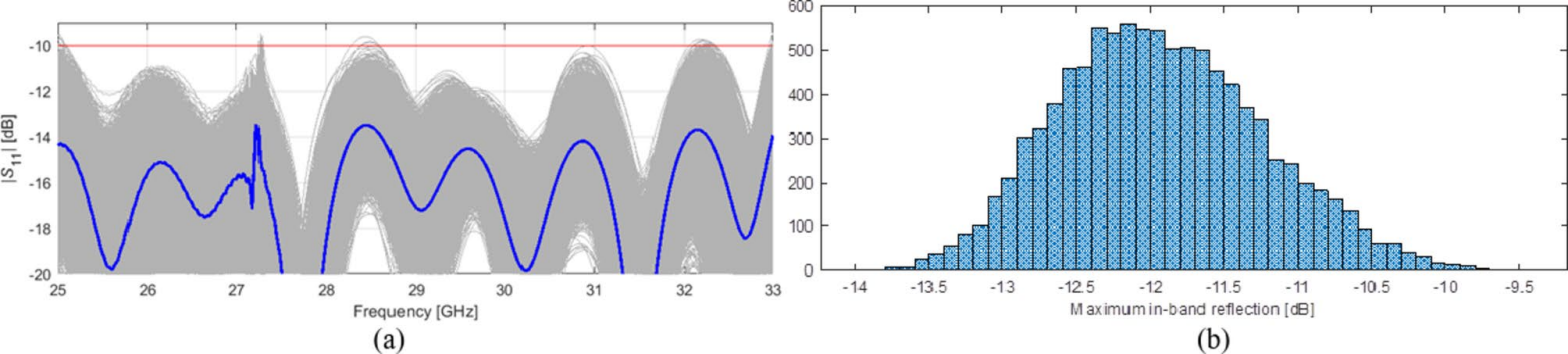
Statistical analysis of the optimized 8 × 8 array assuming maximum geometry parameter deviations of 0.03 mm (corresponding to manufacturing tolerances): (**a**) visualization of the Monte Carlo analysis carried out using 10,000 random samples, (**b**) histogram of the maximum in-band reflection level.

The simulated reflection coefficient of the optimized 8 × 8 slot array antenna has been depicted in Fig. 12. As it can be observed, the maximum in-band |S_11_| measures approximately − 15 dB. In order to verify the design robustness, a statistical analysis of the optimized array has been carried out, considering a maximum parameter deviation of 0.03 mm (which is aligned with the manufacturing process tolerances). The results indicate that the likelihood of fulfilling the condition |S11| ≤ − 10 dB is approximately 99.6%. The results of the statistical analysis have been visualized in Fig. 13. It should be noted; however, that practical implementation, there may exist additional sources of error beyond the aleatory uncertainties considered above. Important design parameters after optimization are summarize in Table 2.

The current design of the 8 × 8 slot array antenna assumes ideal manufacturing conditions, where each layer is perfectly attached to the other, as depicted in Fig. 10. However, practical implementation introduces mechanical tolerances that may lead to leakage. To mitigate potential losses, we incorporated a single pin/hole line providing a minimum of 30 dB isolation^25^. The revised design of the 8 × 8 slot array antenna is outlined below.

### ***Pin/Hole Line Selection***

**Fig. 14:**
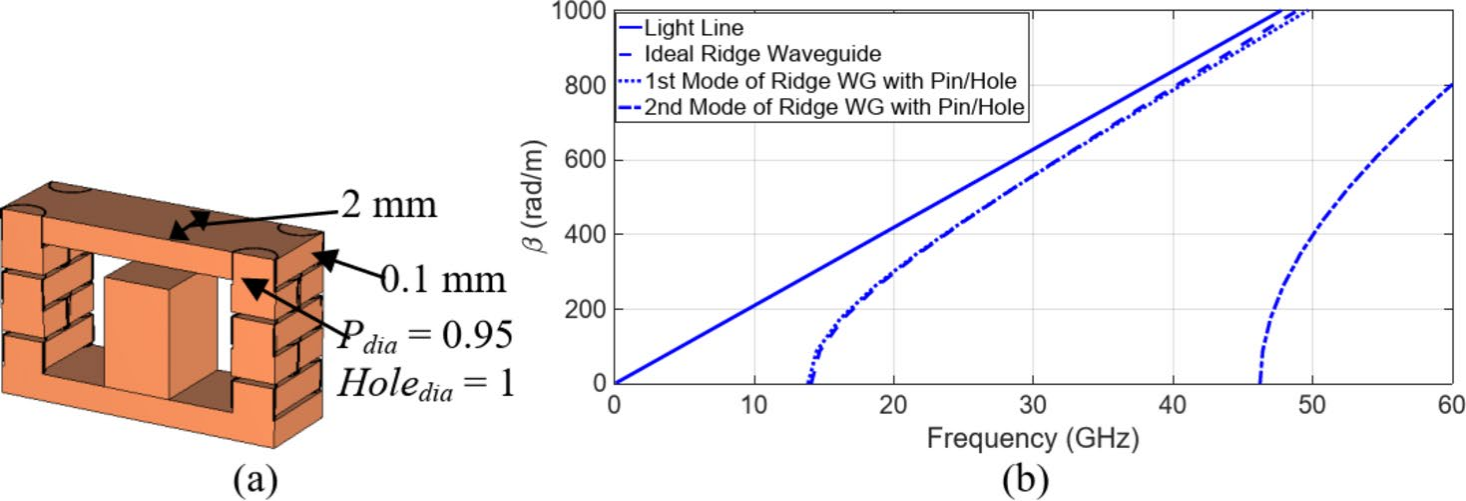
Configuration of the six layer pin/hole layers. (**a**) detailed dimensions of the periodic pin/hole geometry along with the ridge, (**b**) dispersion diagram of the unit cell and its comparison with the ideal ridge waveguide of the same dimension.

The pin-hole arrangement along the transmission line performs similarly to the via holes in substrate integrated waveguides (SIWs). The spacing between adjacent pins and corresponding holes is determined using the principles outlined in^26^. Unlike conventional rectangular waveguides, SIW structures can experience leakage due to periodic gaps, as indicated in^25^. This results in wave propagation characteristics that differ from those of standard rectangular waveguides, including the presence of certain leakage modes. To address this, the modes propagating along the periodic structure’s main transmission line are analyzed using the Eigenmode solver in CST Microwave Studio. This analysis helps establish the operating bandwidth of the transmission line based on the specified pin-hole diameter and spacing. Based on the study presented in^25^, the first step involved determining the dimensions of the pins/holes and their required periodicity to emulate a multi-layer pins/holes ridge waveguide closely resembling an ideal ridge waveguide featuring the same specifications. Through parametric analysis, we identified the optimal dimensions for the pins and holes as 0.95 mm and 1 mm in diameter, respectively, with a 1 mm distance between two consecutive pins, as shown in Fig. 14(a). Analysis of the dispersion diagram shown in Fig. 14(b) indicates that the first mode of the pin/hole ridge waveguide closely resembles that of an ideal ridge waveguide. Notably, the appearance of the second mode within the pin/hole structure begins beyond 45 GHz. Consequently, these dimensions align well with our requirements for the slot array antenna.

**Table 2:** Important design parameters of 8 × 8 element array.

Parameters	Value [mm]
*W*_*GW*_	4.32
*W*_*Gl*_	8.61
*W*_*GH*_	13.25
*Pin*_*l*_	2.3
*Pin*_*h*_	2.16

#### ***Coupling***

**Fig. 15:**
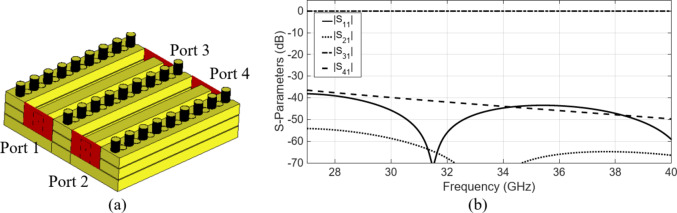
Analysis of mutual coupling between parallel pin/hole waveguides (a) geometry of the two waveguides placed side by side with one row of pin/hole placed in between. (b) S-parameters of the two waveguides placed side by side.

In our proposed technology, the waveguides lack ideal shielding, prompting a thorough assessment of the leakage or coupling between waveguides sharing a common side. This investigation focuses on the mutual coupling between two adjacent waveguides, employing multiple layers with an air gap, featuring a single row of pin/holes positioned between them, as illustrated in Fig. 15(a). For this configuration, the distance between the waveguides is 2.85 mm. As depicted in Fig. 15(b), the introduction of a single row of pin/holes at the multi-layer gaps position (Fig. 15(a)) notably diminishes the coupling between the transmission lines, thereby ensuring a reduction to approximately − 40 dB.

## 8 × 8 slot array with pin/hole

**Fig. 16:**
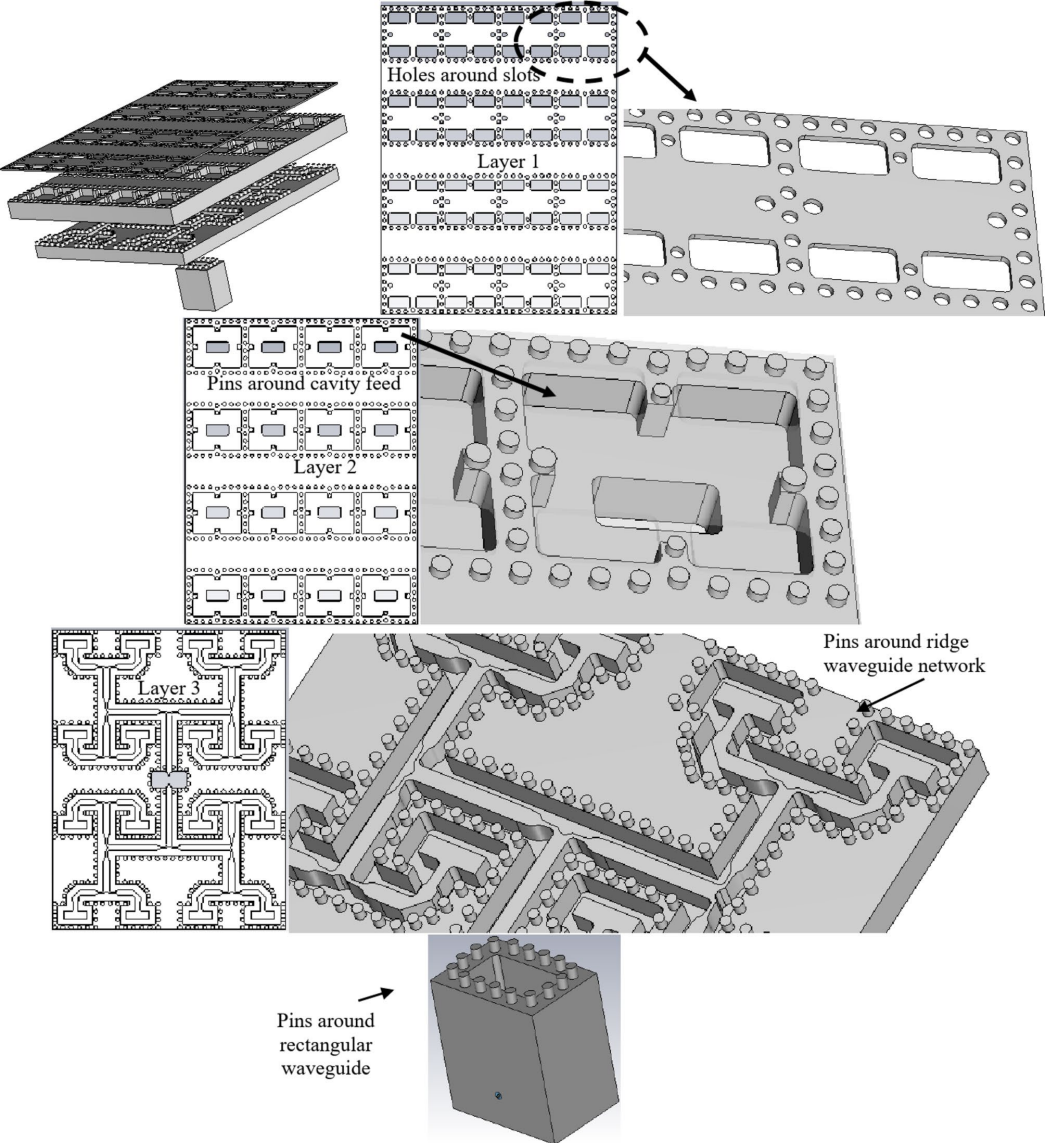
Detailed design and various views of the corporate-fed 8 × 8 slot array antenna with pins/holes.

Figure 16 shows the detailed geometric configuration of the 8 × 8 element slot array antenna. The slots, cavity feed, ridge feed network, vertical rectangular waveguide, and the coaxial feed position have the same dimensions as depicted in the previous sections. As established in the preceding section, the average determined dimensions for pin/hole diameter and spacing are 1 mm, although certain adjustments are made in specific areas due to geometric constraints to occupy the available space. Figure 16 illustrates the arrangement of pins encircling the vertical rectangular waveguide, each corresponding to drilled holes on the rear side of the ridge feed network. Likewise, pins encircle the ridge feed network, mirroring equivalent holes on the rear side of the cavity feed. Finally, the pins placed around the cavity feed align with holes surrounding the slot array. The simulation assumed a 0.1 mm gap between each layer.

**Fig. 17:**
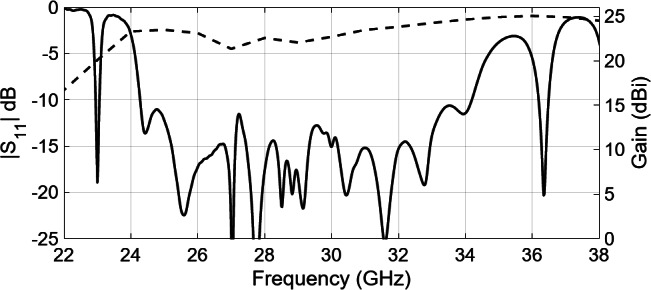
Simulated reflection coefficient and gain of optimized 8 × 8 slot array antenna with pins/holes.

**Fig. 18:**
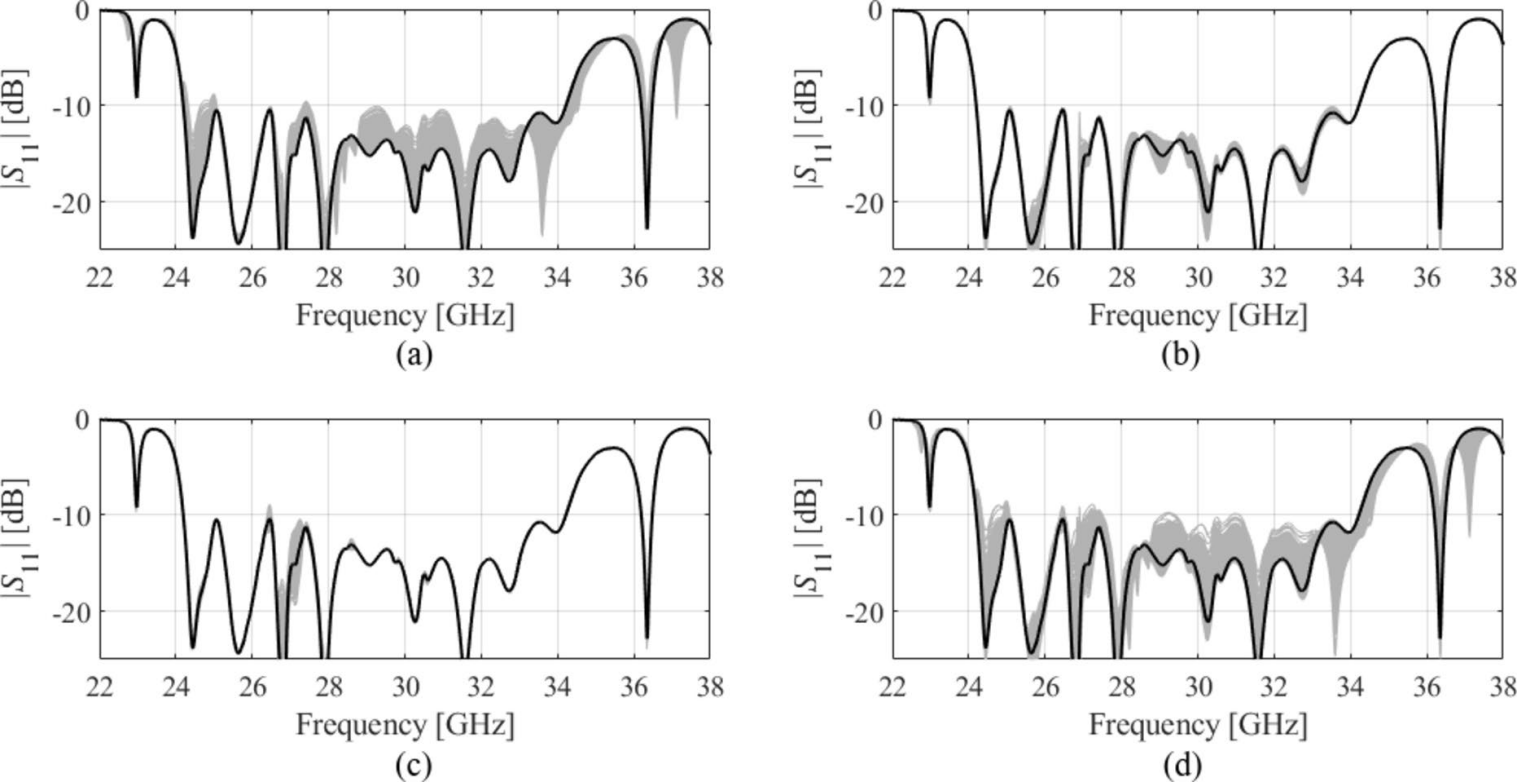
Statistical analysis of antenna’s reflection response variations due to misalignment of the system assembly. Shown are the nominal design (black) and EM-simulated random realizations corresponding to the Monte Carlo simulation (1000 samples, gray): (**a**) the effect of the air gap, (**b**) the effects of *x*-direction misalignments, (**c**) the effect of *y*-direction misalignments, (**d**) collective effects of the air gap and lateral misalignments. The assumed tolerances follow an independent Gaussian distribution with zero mean and 10 μm variance (maximum deviation of 35 μm).

Figure 17 presents the simulated reflection coefficient, |S_11_| of the array. The comparison reveals that the simulated |S_11_| closely aligns with the performance of the ideal 8 × 8 element slot array antenna depicted in Fig. 12. Figure 17 also shows the simulated broadside gain of the array. The gain consistently reaches approximately 23 dBi across most of the operating frequency band. A prototype comprising 8 × 8 slots has been produced through CNC milling. Multiple viewpoints of the manufactured antenna are shown in Fig. 18. This fabricated antenna exhibits a planar design, featuring an aperture size measuring 64 × 84 mm², and a total thickness of 23 mm, which encompasses the vertical rectangular waveguide as well. It is important to observe that due to the limitations in the fabrication process, all sharp corners and edges have been rounded with minimum radius 0.25 mm.

The performance of the proposed design is partially contingent on the accuracy of assembling the elements. To investigate the effects of assembly imperfections, a statistical analysis was carried out assuming tolerances for lateral allocation of the top and the bottom part of the assembly and a potential air gap between these parts. The tolerances concerning *x*- and *y*-direction misalignment and the airgap *d*_*a*_, are assumed to follow independent Gaussian distribution with zero mean and variance of 10 μm (only the positive part of the distribution was assumed for the air gap, which obviously cannot take negative values), with the maximum deviation of 35 μm. Due to low levels of the tolerances, a linear expansion model was used to represent the antenna’s reflection coefficient, which takes the form of *S*_11_(***x***,*f*) = *S*_11_(***x***^(0)^,*f*) + ∇*S*_11_(***x***^(0)^,*f*)(***x*** – ***x***^(0)^), where ***x*** is the actual design parameter vector, ***x***^(0)^ is the nominal (optimized) antenna design parameter vector, and *f* is the frequency. The response gradients are obtained through finite differentiation regarding the aforementioned factors (the air gap and lateral misalignments). The perturbation size corresponds to the maximum deviation of 35 μm.

**Fig. 19:**
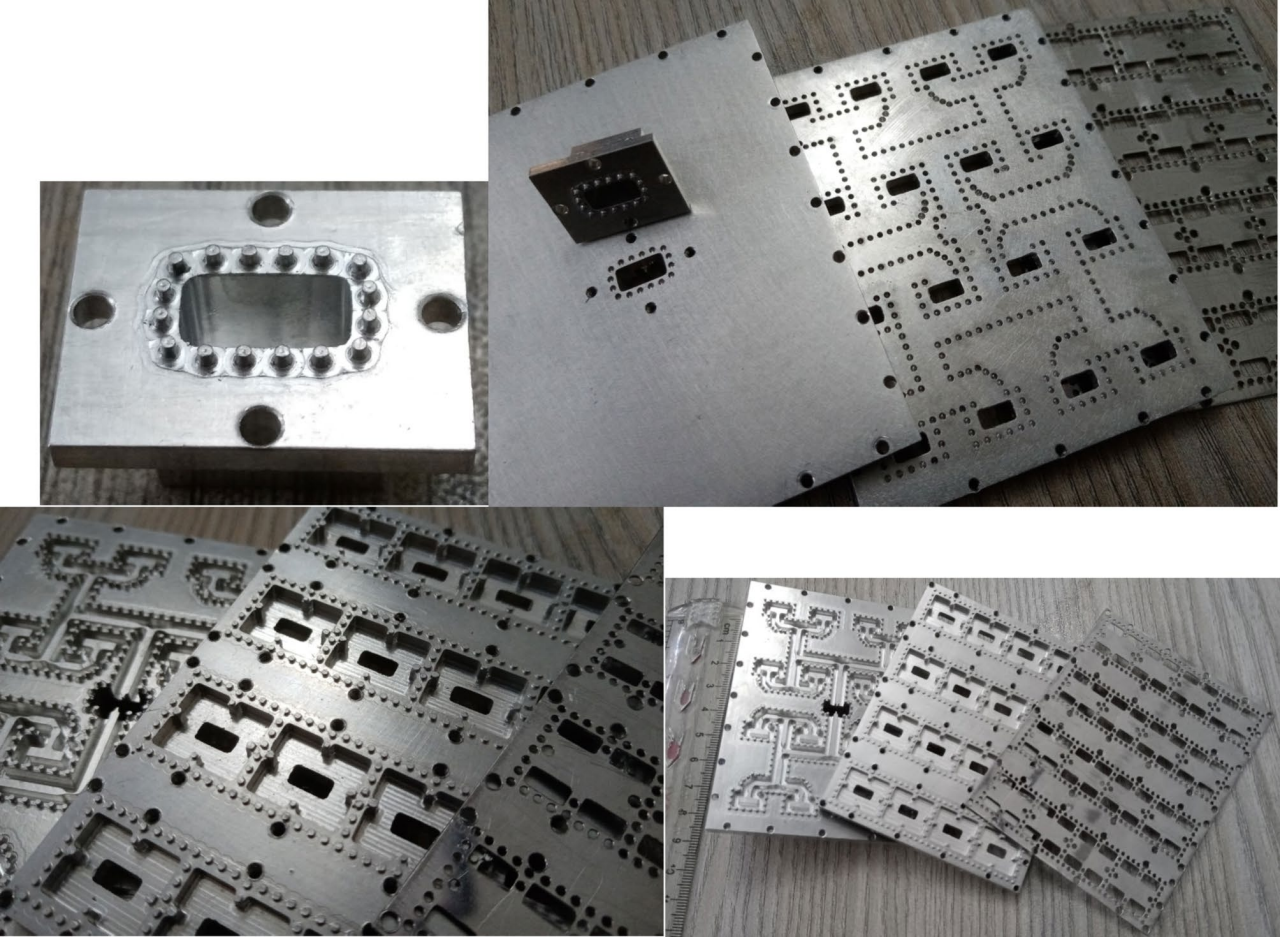
Manufactured prototype of the 8 × 8 slot array antenna with pins/holes.

A Monte Carlo simulation has been performed using the above linear model, illustrated in Fig. 19. As observed, the air gap has the most significant effects on |*S*_11_|. In contrast, the impact of lateral misalignments is considerably smaller. The fabrication yield (the percentage of random realizations for which |*S*_11_| ≤ − 10 dB over the frequency range 25 GHz to 33 GHz) is 81% (air gap), 100% (*x*-direction misalignments), 83% (*y*-deviation misalignments), and 59% (collective misalignments). These results indicate the design’s robustness concerning mechanical assembly imperfections.

## 8 × 8 Slot array antenna performance

**Fig. 20:**
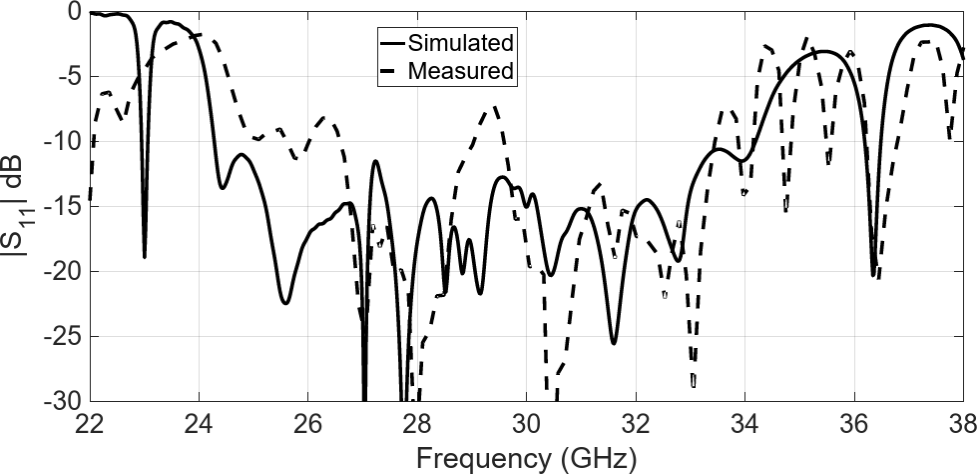
Simulated and measured reflection coefficient of 8 × 8 slot array antenna with Pins/holes.

The reflection coefficient |S_11_| of the fabricated array antenna underwent measurement using a Vector Network Analyzer connected to the 2.92 mm coaxial input port. Figure 20 shows the comparison between the measured and simulated input reflection coefficients. Notably, the fabricated antenna maintains a reflection coefficient |S_11_| below − 10 dB across the frequency range of 25–35 GHz. While differences exist between the simulated and measured results primarily attributed to manufacturing and assembly tolerances (cf. Figure 13), the overall agreement prevails.

**Fig. 21:**
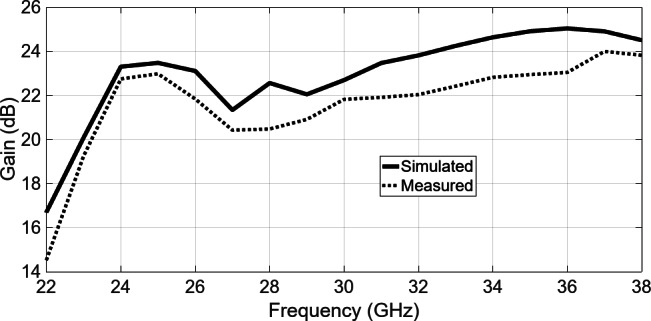
Simulated and measured gain of fabricated 8 × 8-element array antenna.

**Fig. 22:**
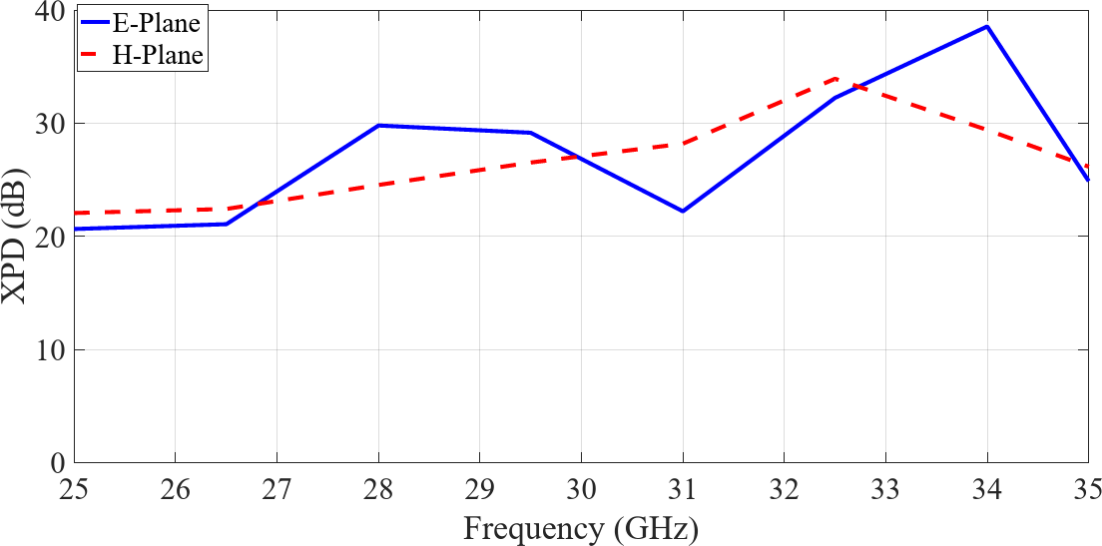
Measured cross-polar decoupling of fabricated 8 × 8-element array antenna, both in E- and H-Plane.

Radiation patterns of the array antenna and gains were measured within an anechoic chamber. Figure 21 shows the simulated and measured frequency characteristics of the realized boresight gain. Dotted line shows the measured gain for an aperture of 64 × 84 mm². The measured gain exceeds 22 dBi on average. Demonstrating a fairly flat response, the gain variation remains within 2 dB over the 25–35 GHz bandwidth. The measured mean realized gain across the operational bandwidth exceeds 22 dBi, with a gain-to-directivity difference of approximately 1.5 dB. This indicates an antenna efficiency of nearly 70% across the entire bandwidth. A degradation in gain is observed around 27 GHz, which may be attributed to input mismatch at this specific frequency, resulting in reduced efficiency and corresponding gain reduction. Figure 22presents the measured cross-polar decoupling, also referred to as cross-polar isolation, across the entire frequency range. The results demonstrate an XPD exceeding 20 dB in both the E-plane and H-plane. The formula used to calculate XPD is provided in^38^ and is detailed below

$$\:{\left(XPD\right)}_{dB}=10log{\left|\frac{{E}_{co}}{{E}_{xp}}\right|}^{2}$$

**Fig. 23:**
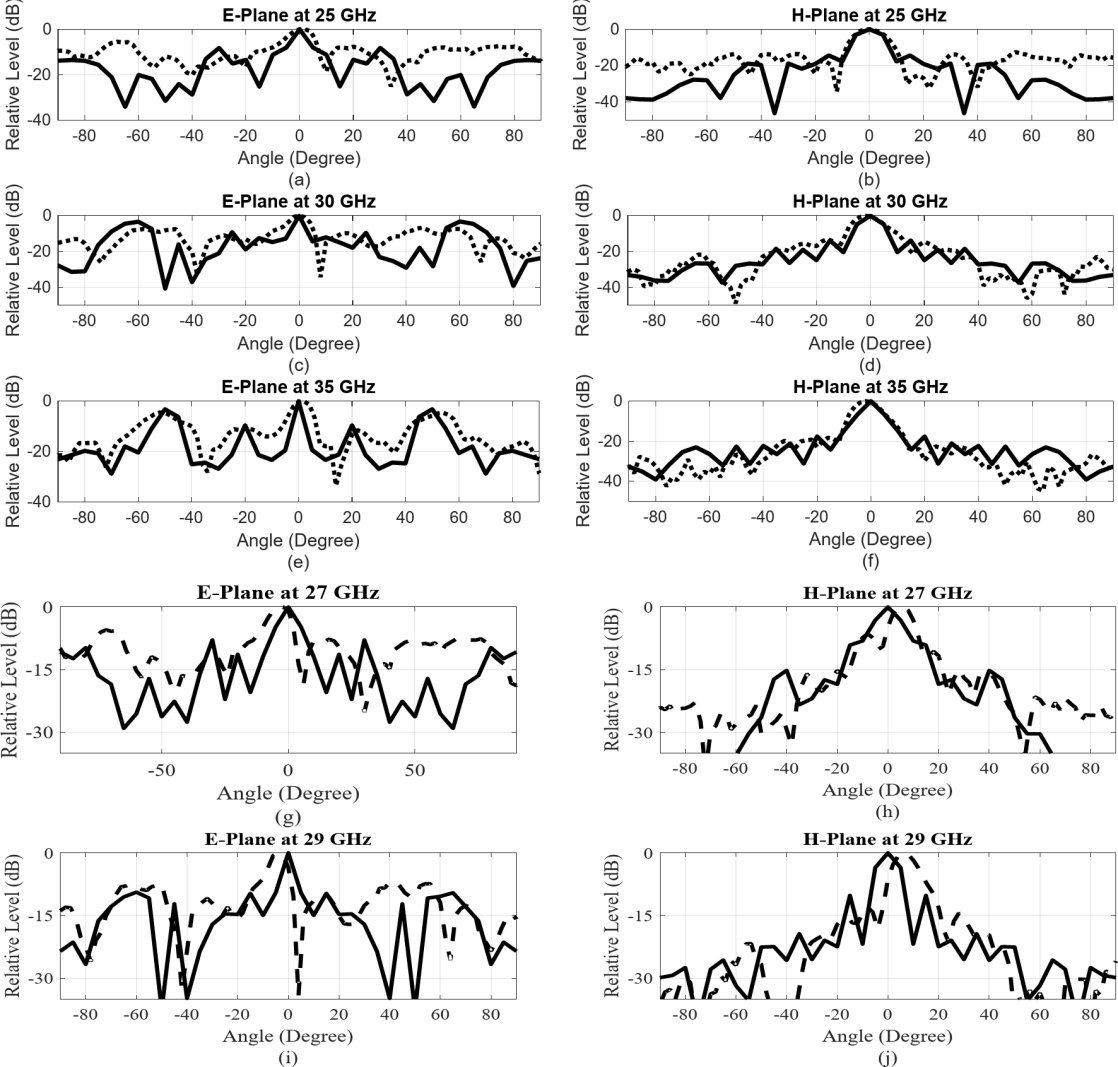
Simulated (solid) and measured (dashed) radiation patterns of the fabricated array at E- and H-plane at frequencies 25, 27, 29, 30 and 35 GHz.

Discrepancies between the measured and simulated gain could arise from additional reflection and ohmic losses attributable to surface roughness affecting metal conductivity. Figure 23 shows both the simulated and measured radiation patterns at 25, 30, and 35 GHz across the E- and H-planes. In the E-plane, the sidelobe levels were measured at approximately − 9 dB. We acknowledge that there are notable radiations at around ± 60° in the E-plane, which could indicate potential grating lobes. Given the slot spacing in the E-plane, which approaches one wavelength at 30 GHz, this spacing could indeed introduce grating lobes, contributing to the reduced overall efficiency. This spacing is added due to the feed network designed in Layer 3, underneath the cavity slots. First sidelobe levels in H-plane is around − 18 dB throughout the desired frequency band. Antenna parameters at the selected frequency points are summarized in Table 3. The larger discrepancies in the H-plane radiation pattern compared to the E-plane are primarily due to a combination of mechanical tolerances, material losses, and measurement setup effects. The antenna was fabricated using aluminum alloy via metal milling techniques, with a machining precision tolerance of 30 to 50 μm. Small variations in the slot dimensions and positioning can introduce phase errors, which affect the H-plane more significantly due to the phase-sensitive nature of the wave propagation in this plane.

Additionally, the choice of a lower-grade aluminum alloy resulted in increased conductor losses, which impacts the efficiency and gain of the antenna, particularly in the H-plane where the current distribution is more affected. Furthermore, the measurement setup may introduce discrepancies, such as cable positioning, connector losses, and reflections from surrounding objects, which tend to have a more noticeable impact on the H-plane due to polarization and scattering effects.

In contrast, the observed variations in the E-plane are primarily attributed to side lobes, which arise from the slot spacing along the E-plane being close to one free-space wavelength. This design decision, influenced by the use of wider ridge waveguide lines for equal-phase feeding, affects the radiation pattern but does not contribute as significantly to the discrepancies between simulation and measurement as seen in the H-plane.

 To further investigate the observed differences, additional simulations incorporating fabrication tolerances (summarized in Fig. 19, Sect. 4, sub-section “8 × 8 Slot Array with Pin/Hole”) could provide a more comprehensive understanding.

**Table 3:** Summary of antenna performance.

Parameter	Value				
Frequency (GHz)	25	27.5	30	32.5	35
*Simulated Directivity (dBi)*	24.64	22.35	23.3	24.3	25.22
*Simulated Gain (dBi)*	23.48	21.9	22.7	24.03	24.91
*Measured Gain (dBi)*	22.98	20.43	21.82	22.2	22.94
*Simulated E-Plane SLL*	−9.2	−8.4	−7.1	−7.3	−8.1
Measured E-Plane SLL	−9.0	−8.7	−8.7	−8.0	−7.0
Simulated H-Plane SLL	−18.2	−18.0	−18.5	−19.1	−19.5
*Measured H-Plane SLL*	−18.1	−17.8	−18.4	−19.04	−19.45

**Table 4:** Comparison with state-of-the-art antenna arrays.

Reference	f_o_ (GHz)	Slots	Layers	Size (λ_o_)	BW (%)	Gain (dBi)	SLL (dB)	e_rad_(%)	Type
^4^	41.3	16	4	3.48 × 3.48	18.9	20.4	−9.1	79.4	ME-dipole
^31^	60	144	1		4.16	22	−15	68	SIW/Mstrip
^32^	60	50	1	7 × 7	12.5	25.2	−9	63.7	Mstrip
^33^	20	256	2	12 × 12.6	15	29.1	−17	76	SIW
^34^	30	64	12	6 × 7.6	13.3	22.5	−12	57.8	Mstrip
^35^	12.5	64	2	6.3 × 6.3	13	24	−12		SIW/Mstrip
^36^	30	64	2	7.2 × 7.7	11.1	23.5	−11.7	79.4	PMC + Mstrip
^7^	61	256	3	15.2 × 15	13	33		82.5	WG
^37^	62.5	64	2	13 × 13	19	32		65	RGW
This Work	30	64	3	6.4 × 8.4	33	23	−10	71	RW

.

## Conclusion

In this study, we developed and experimentally validated a high-gain slot antenna array with broad bandwidth capabilities optimized for operation at 30 GHz. Our approach incorporated a corporate feed network built on ridge waveguide technology. Main objective of this research work is to demonstrate another possible way of shielding the cavity circuits. Shielding is achieved by placing pins/holes around slots, ridges and cavity feed, eliminated the necessity for electrical contact among different layers within a multilayer antenna structure. To simplify the antenna excitation process, double transition structures facilitating seamless connections between the ridge waveguide, rectangular waveguide, and the 2.92 mm coaxial connector have been presented. Additionally, our novel design introduces mechanical support for the upper, extremely thin slot layer using a pin/hole texture. This innovation culminated in the design, simulation, fabrication, and measurement of an 8 × 8-element array. Table 4 illustrates a comparative analysis among various studies conducted in different technologies and within similar frequency bands. From Table 4, it can be seen that our design has more wideband in nature and smaller in size, especially with gap waveguide technology. The measured realized gain is about 22 dBi across the entire operational bandwidth spanning 25 to 35 GHz, representing a remarkable 33% bandwidth.

## Data Availability

All data has been included in the study.
